# Key Players and Individualists of Cyclic-di-GMP Signaling in *Burkholderia cenocepacia*

**DOI:** 10.3389/fmicb.2018.03286

**Published:** 2019-01-10

**Authors:** Anja M. Richter, Mustafa Fazli, Nadine Schmid, Rebecca Shilling, Angela Suppiger, Michael Givskov, Leo Eberl, Tim Tolker-Nielsen

**Affiliations:** ^1^Costerton Biofilm Center, Department of Immunology and Microbiology, Faculty of Health and Medical Sciences, University of Copenhagen, Copenhagen, Denmark; ^2^Department of Microbiology, University of Zurich, Zurich, Switzerland; ^3^Singapore Centre for Environmental Life Sciences Engineering, Nanyang Technological University, Singapore, Singapore

**Keywords:** Cyclic-di-GMP, *Burkholderia cenocepacia*, biofilm formation, motility, RpfR, Bcal2449, GGDEF EAL domain proteins

## Abstract

*Burkholderia cenocepacia* H111 is an opportunistic pathogen associated with chronic lung infections in cystic fibrosis patients. Biofilm formation, motility and virulence of *B. cenocepacia* are regulated by the second messenger cyclic di-guanosine monophosphate (c-di-GMP). In the present study, we analyzed the role of all 25 putative c-di-GMP metabolizing proteins of *B. cenocepacia* H111 with respect to motility, colony morphology, pellicle formation, biofilm formation, and virulence. We found that RpfR is a key regulator of c-di-GMP signaling in *B. cenocepacia*, affecting a broad spectrum of phenotypes under various environmental conditions. In addition, we identified Bcal2449 as a regulator of *B. cenocepacia* virulence in *Galleria mellonella* larvae. While Bcal2449 consists of protein domains that may catalyze both c-di-GMP synthesis and degradation, only the latter was essential for larvae killing, suggesting that a decreased c-di-GMP level mediated by the Bcal2449 protein is required for virulence of *B. cenocepacia*. Finally, our work suggests that some individual proteins play a role in regulating exclusively motility (CdpA), biofilm formation (Bcam1160) or both (Bcam2836).

## Introduction

Cyclic di-guanosine monophosphate (c-di-GMP) is an ubiquitous intracellular second messenger, regulating motility, biofilm formation, cell differentiation and virulence in numerous bacteria and some Dictyostelia (Chen and Schaap, 2012; Römling et al., 2013). It is synthesized out of two molecules of GTP by diguanylate cyclases (DGCs) with conserved GGDEF-domains as enzymatically active sites. Phosphodiesterases (PDEs) with conserved EAL- or HD-GYP-domains are required for converting c-di-GMP into pGpG, while further hydrolysis into GMP is catalyzed by some PDEs or by additional oligoribonucleases (Hengge, 2009; Römling et al., 2013; Orr et al., 2015; Jenal et al., 2017). Cyclic-di-GMP metabolizing domains are often coupled with sensor domains, enabling the cell to react to intracellular or environmental changes with modified c-di-GMP levels through modulation of DGC or PDE activities (Galperin, 2004). Once synthesized, c-di-GMP binds to a number of receptor molecules, e.g., enzymes, transcription factors and riboswitches, or it stabilizes multi-protein-complexes (Hengge, 2009; Ryan et al., 2012; Römling et al., 2013). Equally diverse are the regulated target molecules and phenotypic outputs. High c-di-GMP levels are known to decrease single-cell motility through inhibition of flagellar gene expression and rotation, and also down-regulate synthesis of acute virulence factors. Moreover, high c-di-GMP levels stimulate the production of extracellular matrix substances, and thereby positively regulate biofilm formation (Ryjenkov et al., 2006; Hickman and Harwood, 2008; Boehm et al., 2010; Baraquet et al., 2012; Hall and Lee, 2018). Biofilms in the host can often resist antibiotic treatments and serve as permanent pathogen reservoirs, often leading to chronic infections (Parsek and Singh, 2003; Ciofu et al., 2015). Due to the functional redundancy of c-di-GMP-forming, -binding and -degrading proteins, a theory of multiple c-di-GMP regulation modules acting in parallel or on subsequent hierarchic levels has been proposed recently (Kader et al., 2006; Povolotsky and Hengge, 2012; Sarenko et al., 2017; Dahlstrom et al., 2018). Within those modules, only one or a few DGCs and PDEs regulate a specific effector and target molecule, thereby affecting only one cellular output. For example, some c-di-GMP-metabolizing proteins, such as AdrA and DgcC in *Salmonella* and *Escherichia coli* or FimX in *P. aeruginosa*, regulate a single specific phenotype, in this case cellulose biosynthesis and twitching motility, respectively (Zogaj et al., 2001; Brombacher et al., 2003; Kazmierczak et al., 2006). In contrast, global regulators, such as PdeR in *E. coli*, BldD in *Streptomyces* or PleD in *C. crescentus*, affect multiple c-di-GMP-controlled processes (Paul et al., 2004; Lindenberg et al., 2013; Tschowri et al., 2014).

The *Burkholderia cepacia* complex (*Bcc*) consists of 22 species (Vanlaere et al., 2009; De Smet et al., 2015; Depoorter et al., 2016; Bach et al., 2017; Martina et al., 2018), often associated with infections in plants, animals and humans. Besides the *Bcc*, the *Burkholderia* genus also includes *B. pseudomallei* and *B. mallei*, the causative agents of melioidosis and glanders in humans and animals, respectively. Over the past decades, the *Bcc* member *B. cenocepacia* has emerged as an important opportunistic human pathogen for immunocompromised individuals and patients suffering from cystic fibrosis (CF) (Mahenthiralingam et al., 2005; Chiarini et al., 2006). Within the CF lung, *B. cenocepacia* infections cause severe complications, known as the cepacia-syndrome, including decline in lung function and increased mortality (Drevinek and Mahenthiralingam, 2010; Bragonzi et al., 2012). *B. cenocepacia* can produce several biofilm matrix components, including the exopolysaccharides Bep, cellulose, cepacian and poly-β-1,6-*N*-acetylglucosamine, proteinaceous compounds, such as BapA, lectins and type-1 fimbria, and extracellular DNA (Conway et al., 2004; Chiarini et al., 2006; McCarthy et al., 2010; Fazli et al., 2011, 2012; Yakandawala et al., 2011; Inhulsen et al., 2012; Messiaen et al., 2014).

Similar to many other Gram-negative bacteria, the production of extracellular matrix components and the resulting formation of pellicles and flow-cell biofilms in *Burkholderia* sp. are shown to be positively regulated by c-di-GMP; whereas motility is negatively regulated by the second messenger (Lee et al., 2010; Fazli et al., 2011, 2012, 2014; Plumley et al., 2017; Kumar et al., 2018). However, in contrast to the situation in, e.g., *P. aeruginosa*, where c-di-GMP stimulates biofilm formation in microtiter plates (Hickman et al., 2005m et al., 2015.), its role in this specific form of biofilm formation in the members of the genus *Burkholderia* is unclear, and contradicting results have been reported (Lee et al., 2010; Deng et al., 2012; Jung et al., 2017; Plumley et al., 2017; Schmid et al., 2017). Prior work in our group has suggested that synthesis of the exopolysaccharide Bep is positively regulated by c-di-GMP and the two transcriptional regulators BerA and BerB (Fazli et al., 2011, 2012, 2017). Moreover, it is known that the *cis*-2-dodecenoic acid-based quorum sensing system (referred to as the *Burkholderia* diffusible signal factor, BDSF, in *Burkholderia* spp.) stimulates c-di-GMP degradation via RpfR, a BDSF-binding GGDEF-EAL domain protein, and thereby regulates virulence in *B. cenocepacia* (Deng et al., 2012, 2013; Schmid et al., 2017; Yang et al., 2017). Point mutations in the BDSF-binding domain of RpfR have been reported in *B. multivorans* strains isolated from patients with chronic lung infections (Silva et al., 2016), highlighting the role of c-di-GMP in chronic infections not only in *B. cenocepacia*, but also in other related *Burkholderia* spp.

In the present study, we investigated individual mutants of all 25 GGDEF-/EAL-/HD-GYP-domain protein encoding genes present in *B. cenocepacia* H111 for their roles in the regulation of multiple c-di-GMP-dependent phenotypes. We found that RpfR acts as a key regulator in various c-di-GMP signaling-dependent processes, we present evidence that the Bcal2449 protein is a regulator of virulence, and we identified network-components with specific effects on a limited number of c-di-GMP regulated processes.

## Materials and Methods

### Bacterial Strains and Growth Conditions

Bacterial strains and plasmids used in this study are listed in Tables 1, 2, respectively. Unless stated otherwise, *B. cenocepacia* and *E. coli* strains were incubated at 37°C. Lysogeny Broth (LB) was used for overnight cultivation, and it was supplemented with antibiotics when necessary at the following concentrations: 25 μg/ml gentamicin (Gm), 50 μg/ml kanamycin (Km), 80 μg/ml (liquid medium) or 120 μg/ml (solid medium) tetracycline (Tet), 100 μg/ml trimethoprim (Tp) for *B. cenocepacia* strains; 100 μg/ml ampicillin (Amp), 6 μg/ml chloramphenicol (Cm), 10 μg/ml Gm, 50 μg/ml Km, 20 μg /ml Tet, 50 μg/ml Tp for *E. coli* strains. After conjugations, bacteria were incubated on LB medium supplemented with appropriate antibiotics and Amp or on AB medium (Clark and Maaløe, 1967) supplemented with 10 mM Na-citrate as carbon source to inhibit *E. coli* growth and select for *B. cenocepacia* transconjugants. When indicated, salt-free medium (LBnoNaCl or ABnoNaCl) was used for biofilm analysis.

**Table 1:** Bacterial strains used in this study.

Bacterial strain	Characteristics	Reference
*B. cenocepacia* H111	Clinical isolate from a cystic fibrosis patient	Gotschlich et al., 2001; Carlier et al., 2014
*B. cenocepacia* H111 *ΔrpfR*	*rpfR* gene deletion mutant	Deng et al., 2012
*B. cenocepacia* H111 *cdpA*	Insertional mutant with *cdpA* interrupted by pEX18Gm, Gm^r^	This study
*B. cenocepacia* H111 *Δbcal0430*	*bcal0430* gene deletion mutant	
*B. cenocepacia* H111 *Δbcal1635*	*bcal1635* gene deletion mutant	
*B. cenocepacia* H111 *Δbcal1975*	*bcal1975* gene deletion mutant	
*B. cenocepacia* H111 *Δbcal2852*	*bcal2852* gene deletion mutant	
*B. cenocepacia* H111 *Δbcam0748*	*bcam0748* gene deletion mutant	
*B. cenocepacia* H111 *Δbcam1161*	*bcam1161* gene deletion mutant	
*B. cenocepacia* H111 *Δbcam1554*	*bcam1554* gene deletion mutant	
*B. cenocepacia* H111 *Δbcam1670*	*bcam1670* gene deletion mutant	
*B. cenocepacia* H111 *Δbcam2256*	*bcam2256* gene deletion mutant	
*B. cenocepacia* H111 *Δbcam2822*	*bcam2822* gene deletion mutant	
*B. cenocepacia* H111 *Δbcam2836*	*bcam2836* gene deletion mutant	
*B. cenocepacia* H111 *Δbcas0398*	*bcas0398* gene deletion mutant	
*B. cenocepacia* H111 *Δbcal0621*	*bcal0621* gene deletion mutant	
*B. cenocepacia* H111 *Δbcal2449*	*bcal2449* gene deletion mutant	
*B. cenocepacia* H111 *Δbcam1160*	*bcam1160* gene deletion mutant	
*B. cenocepacia* H111 *Δbcal0652*	*bcal0652* gene deletion mutant	
*B. cenocepacia* H111 *Δbcal1100*	*bcal1100* gene deletion mutant	
*B. cenocepacia* H111 *Δbcal2749*	*bcal2749* gene deletion mutant	
*B. cenocepacia* H111 *Δbcal3188*	*bcal3188* gene deletion mutant	
*B. cenocepacia* H111 *Δbcam0158*	*bcam0158* gene deletion mutant	
*B. cenocepacia* H111 *Δbcam2426*	*bcam2426* gene deletion mutant	
*B. cenocepacia* H111 *Δbcas0263*	*bcas0263* gene deletion mutant	
*B. cenocepacia* H111 *Δbcas0378*	*bcas0378* gene deletion mutant	
*E. coli* DH5α	Used for standard DNA manipulations	Invitrogen
*E. coli* DB3.1	Host for Gateway-compatible gene replacement vectors	Invitrogen
*E. coli S17-1-βpir*	Host of the mini-Tn7-kan-*gfp* delivery vector	Simon et al., 1983

**Table 2:** Plasmids used in this study.

Plasmid	Characteristics	Reference
pEX18Gm	Suicide vector for *cdpA* mutant construction	Hoang et al., 1998
Pmini-Tn7-*kan*-*gfp*	Delivery vector for mini-Tn7-kan-gfp, Km^r^	Norris et al., 2010
pRK600	Helper plasmid in tri- and four-parental matings; *ori*-ColE1 RK-*mob*^+^ RK-*tra*^+^; Cm^r^	Kessler et al., 1992
pUX-BF13	Helper plasmid providing the Tn7 transposition functions in trans; *mob*+ *ori*-R6K; Amp^r^	Bao et al., 1991
pDAI-SceI-*pheS*	Cloning vector containing the I-SceI endonuclease and *pheS*; Tet^r^	Fazli et al., 2015
pDONRPEX18Tp-SceI-pheS	Gateway compatible gene replacement vector based on SceI and pheS; Tp^r^	Fazli et al., 2015
pDONRPEX18Gm-SceI-pheS	Gateway compatible gene replacement vector based on SceI and pheS; Gm^r^	Fazli et al., 2015
pENTRPEX18Tp-SceI-*pheS*-*bcal0430*	Gene replacement vector containing the *bcal0430* deletion allele, Tp^r^	This study
pENTRPEX18Gm-SceI-*pheS*-*bcal1635*	Gene replacement vector containing the *bcal1635* deletion allele, Gm^r^	
pENTRPEX18Tp-SceI-*pheS*-*bcal1975*	Gene replacement vector containing the *bcal1975* deletion allele, Tp^r^	
pENTRPEX18Gm-SceI-*pheS*-*bcal2852*	Gene replacement vector containing the *bcal2852* deletion allele, Gm^r^	
pENTRPEX18Tp-SceI-*pheS*-*bcam0748*	Gene replacement vector containing the *bcam0748* deletion allele, Tp^r^	
pENTRPEX18Tp-SceI-*pheS*-*bcam1161*	Gene replacement vector containing the *bcam1161* deletion allele, Tp^r^	
pENTRPEX18Tp-SceI-*pheS*-*bcam1554*	Gene replacement vector containing the *bcam1554* deletion allele, Tp^r^	
pENTRPEX18Tp-SceI-*pheS*-*bcam1670*	Gene replacement vector containing the *bcam1670* deletion allele, Tp^r^	
pENTRPEX18Tp-SceI-*pheS*-*bcam2256*	Gene replacement vector containing the *bcam2256* deletion allele, Tp^r^	
pENTRPEX18Tp-SceI-*pheS*-*bcam2822*	Gene replacement vector containing the *bcam2822* deletion allele, Tp^r^	
pENTRPEX18Tp-SceI-*pheS*-*bcam2836*	Gene replacement vector containing the *bcam2836* deletion allele, Tp^r^	
pENTRPEX18Tp-SceI-*pheS*-*bcas0398*	Gene replacement vector containing the *bcas0398* deletion allele, Tp^r^	
pENTRPEX18Tp-SceI-*pheS*-*bcal0621*	Gene replacement vector containing the *bcal0621* deletion allele, Tp^r^	
pENTRPEX18Tp-SceI-*pheS*-*bcal2449*	Gene replacement vector containing the *bcal2449* deletion allele, Tp^r^	
pENTRPEX18Tp-SceI-*pheS*-*bcam1160*	Gene replacement vector containing the *bcam1160* deletion allele, Tp^r^	
pENTRPEX18Tp-SceI-*pheS*-*bcal0652*	Gene replacement vector containing the *bcal0652* deletion allele, Tp^r^	
pENTRPEX18Tp-SceI-*pheS*-*bcal1100*	Gene replacement vector containing the *bcal1100* deletion allele, Tp^r^	
pENTRPEX18Tp-SceI-*pheS*-*bcal2749*	Gene replacement vector containing the *bcal2749* deletion allele, Tp^r^	
pENTRPEX18Tp-SceI-*pheS*-*bcal3188*	Gene replacement vector containing the *bcal3188* deletion allele, Tp^r^	
pENTRPEX18Tp-SceI-*pheS*-*bcam0158*	Gene replacement vector containing the *bcam0158* deletion allele, Tp^r^	
pENTRPEX18Tp-SceI-*pheS*-*bcam2426*	Gene replacement vector containing the *bcam2426* deletion allele, Tp^r^	
pENTRPEX18Tp-SceI-*pheS*-*bcas0263*	Gene replacement vector containing the *bcas0263* deletion allele, Tp^r^	
pENTRPEX18Tp-SceI-*pheS*-*bcas0378*	Gene replacement vector containing the *bcas0378* deletion allele, Tp^r^	

### Construction of *B. cenocepacia* H111 Mutants and *gfp*-Fusions

Deletion of 23 of the PDE/DGC genes in *B. cenocepacia* was carried out using a protocol based on an allelic exchange system described previously (Fazli et al., 2015). Sequences of the oligonucleotides used for the construction of the gene replacement vectors are available on request. The *rpfR* (*bcam0580*) mutant used in this study has been described previously (Deng et al., 2012). The *cdpA* (*bcal1069*) mutant was constructed as follows. An internal fragment of *cdpA* was amplified by PCR using the primers 5′-ggatccCGTACGAAATGCACCATGAC and 5′-aagcttCTGCCCGTGGTACACGTAG and inserted as a BamHI/HindIII fragment into the respective sites of the suicide plasmid pEX18Gm (Hoang et al., 1998). The resulting plasmid was transferred by triparental conjugation to *B. cenocepacia* H111 wild type to generate a *cdpA* mutant. The introduction of a chromosomal *gfp*-tag into the *B. cenocepacia* strains for confocal laser scanning microscopy (CLSM) was carried out as described previously (Fazli et al., 2012).

### Motility Assays

Swimming motility was tested on plates containing 0.5% tryptone, 0.5% sodium chloride and 0.3% agar (Pesavento et al., 2008). Three μl of an overnight culture (adjusted to OD_600_ 4.0) was inoculated into the swimming plates and cells were allowed to swim for 10 h at the temperature indicated. Assessment of swarming motility was performed using AB medium plates containing 0.5% glucose, 0.5% casamino acids and 0.6% agar as described previously (Givskov et al., 1998). Three μl of an overnight culture (adjusted to OD_600_ 4.0) was spotted on top of the agar medium and cells were allowed to swarm for up to 48 h. Twitching motility was tested using LB plates containing 1% agar. Cell material from overnight grown colonies was stab inoculated through the LB agar medium, ensuring that bacteria grew and moved between the inner bottom of the petri dish and the agar medium. After 48 h incubation at 37°C, the agar medium was removed carefully and bacteria attached to the petri dish were stained with 0.1% crystal violet (CV) solution to visualize the area covered with bacteria.

### Crystal Violet Biofilm Assay

Attachment to abiotic surfaces was assayed as described in Dahlstrom et al. (2018) using BIOLOG PM-1 nutrient plates for phenotypic microarrays with minor modifications. LB overnight cultures were adjusted to OD_600_ 0.716 in ABnoNaCl, mixed 1.5:100 with ABnoNaCl. Hundred-twenty five μl of the inoculated medium was pipetted up and down in the BIOLOG plate and transferred to a 96-well polystyrene microtiter plate (Thermo Fisher, Cat. Nr. 269787) covered with a lid, and the microtiter plate was incubated for 24 h at 37°C to allow biofilm formation. The following day, attached bacteria were quantified using a standard biofilm protocol (O′Toole and Kolter, 1998) with minor modifications as follows. Bacterial growth was measured at OD_600_, the medium was discarded, and the wells were washed twice with distilled water. To avoid biofilm disruption during the following washing steps, the attached cells were fixed by drying the plates overnight. The wells were stained with 175 μl of 0.1% CV at room temperature for 20 min and subsequently washed twice with distilled water. To quantify biofilm formation, the cells were de-stained with 200 μl 30% acetic acid and absorbance of the CV-acetic acid solution was measured at 590 nm. Based on the results obtained from the BIOLOG plates, 18 substances were selected for further investigation, and biofilm assays were repeated at least three times with 4 replicate wells per strain (the substance concentrations used were: 5 mM citric acid; 10 mM L-arabinose/ L-asparagine/ D-fructose/ L-fucose/ L-glutamine/ L-lactic acid/ D-mannitol/ L-proline; 20 mM galactose/ glycolic acid/ glyoxylic acid/ D-mannose; 50 mM L-glutamic acid/ α-D-lactose/ L-serine/ tyramine; 5 mg/ml mucin from porcine stomach). In addition, ABnoNaCl containing glucose, glycerol or Na-citrate (all at 10 mM) as well as LBnoNaCl was included. CV assays were performed similar to the ones with the BIOLOG plates, however, each well contained 150 μl of medium inoculated to an OD_600_ of 0.01. Upon transfer to 37°C, the plates were covered with a 96-pegs polystyrene lid (Thermo Scientific; Cat. Nr. 445497), referred to here as the “Calgary Biofilm Device” (Ceri et al., 1999), which allows bacterial cells to attach to the microtiter wells and the pegs. Bound CV and therefore biofilm formation on the pegs was quantified and analyzed separately in a second microtiter plate containing 30% acetic acid to de-stain the pegs-biofilms for subsequent absorbance measurements.

### Macrocolony Formation

Five μl of an overnight culture grown in LB was spotted on ABnoNaCl medium supplemented with 1.5% agar. After 5 days of incubation at 37°C photos of the macrocolonies were acquired using a Nikon D3300 SLR camera equipped with a 52 mm Nikon DX SWM Micro 1:1 objective.

### Pellicle Formation

Pellicle formation was assayed as described previously (Fazli et al., 2011) with minor modifications. Three ml ABnoNaCl supplemented with carbon sources as indicated was inoculated 1:100 with overnight-grown *B. cenocepacia* culture, and incubated for up to 5 days at 23°C or 30°C. Pellicle formation was photographed on each day.

### Flow-Cell Biofilm Formation and CLSM Analysis

Cultivation of flow-cell biofilms and optical visualization using CLSM were performed as described previously (Fazli et al., 2011, 2012). *gfp*-tagged strains were used in order to visualize biofilms in flow-cell chambers via CLSM. Microscopic observation of biofilms was performed after 24, 48, and 72 h of growth at 37°C using a Zeiss LSM710 confocal laser scanning microscope equipped with a 63×/1.4 objective and an argon laser. Images were acquired with an AxioCam MRm camera and processed using ZEN (Zeiss, Germany) and IMARIS (Bitplane, Zürich, Switzerland) software. Quantification of biofilm biomass was performed by using COMSTAT software (Heydorn et al., 2000). For COMSTAT analysis, a total of 6 CLSM image stacks were acquired randomly for each strain from flow-cell biofilms. Flow-cell experiments were performed at least twice for each mutant strain along with a wild-type strain control in each individual experiment.

### Virulence Assay

Virulence assays were performed using larvae of the Greater Wax Moth (*Galleria mellonella*), purchased from BioSystems Technologies (Exeter, United Kingdom), and experiments were performed as described previously (Agnoli et al., 2014) with minor modifications. Briefly, LB medium supplemented with antibiotics, when necessary, was inoculated 1:100 with bacterial overnight cultures and incubated with shaking at 37°C for 3–3.5 h to an OD_600_ of 0.4–0.7. Thereafter, 250 μl of bacterial culture was harvested by centrifugation, and the cell pellet was resuspended in 10 mM MgSO_4_ to obtain an OD_600_ of 0.125, corresponding to 4^∗^10^7^ CFU/ml. The cell suspension was further diluted 1:10 in 10 mM MgSO_4_ supplemented with Amp, and 5 μl of the cell suspension (10.000–20.000 CFU) was injected into the second last right proleg of *G. mellonella* larvae using a Hamilton syringe. The syringe was washed with 2.6% NaClO, 70% ethanol, and MgSO_4_ prior to continuing with the next strain. Eight-to-10 larvae were used for each strain tested and experiments were performed at least 3 times per strain with a wild-type control in each experiment. Upon arrival, un-infected larvae were kept at 12°C for up to 10 days and were transferred to room temperature 4 h prior to infection. After injection of the bacteria the larvae were transferred with a small tissue paper into petri dishes and incubated in a plastic box containing wet paper towels at 30°C for up to 4 days. The larvae were observed daily for viability. In parallel, serial dilutions of the remaining inocula were spread on LB-agar plates for CFU counts to verify that each larva was injected with a similar amount of bacterial cells for each strain tested.

## Results

### The *B. cenocepacia* H111 Genome Codes for 25 Putative c-di-GMP-Metabolizing Proteins

**FIGURE 1:**
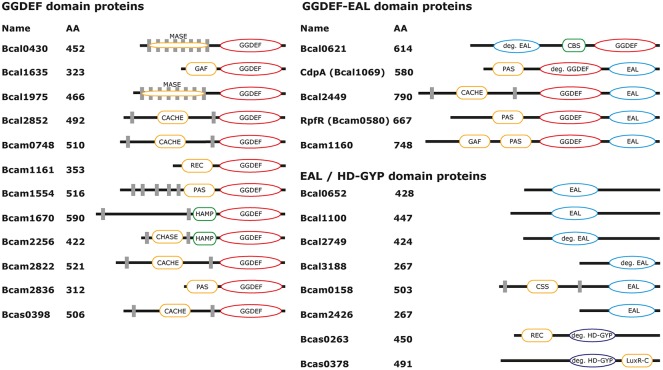
Domain structure of GGDEF/EAL/HD-GYP proteins of *B. cenocepacia* H111. Proteins were identified by comparative BLAST analysis of well described GGDEF-, EAL- and HDY-GYP domains from *E. coli* and *X. campestris* (see text for details). Protein sequences were analyzed with Pfam and NCBI-CDD web tools. Transmembrane domains (gray squares) were verified with the TMHMM web tool. GGDEF domains are highlighted in red, EAL domains in blue and HD-GYP domains in violet, sensor domains in yellow or green. If a protein name has been assigned, it is also shown here.

Comparative BLAST analysis using the GGDEF domain of *E. coli* YdaM [aa241-410], the EAL domain of *E. coli* YjcC [aa275-510] and the HD-GYP domain of *Xanthomonas campestris* pv. Campestris RpfG [aa202-319] revealed that the *B. cenocepacia* H111 genome codes for twenty-five GGDEF, EAL, or HD-GYP domain containing proteins (Figure 1). Based on the presence of amino acid residues known to be required for enzymatic activities and co-factor binding (Rao et al., 2008; Tchigvintsev et al., 2010; Römling et al., 2013), we predicted possible c-di-GMP metabolizing activities. Twelve genes encode proteins with conserved GGDEF domains, and therefore being potential DGCs. Eight genes encode proteins with EAL or HD-GYP domains, four (Bcal0652, Bcal1100, Bcam0158, and Bcam2426) of which have conserved domains and therefore being potential PDEs. Five genes encode composite proteins with both GGDEF and EAL domains. Among the composite proteins two (Bcal0621 and CdpA) contain only one conserved domain, indicating that these proteins most likely function as DGC and PDE, respectively; whereas the three other proteins (Bcal2449, RpfR, and Bcam1160) contain intact GGDEF and EAL domains, indicating that these proteins might be bifunctionally active. The chromosome 1 of *B. cenocepacia* J2315 contains a 57-kb duplication (from bcal0969 to bcal1026 and from bcal2901 to bcal2846), resulting in a gene duplication of *bcal1020* (with the paralog *bcal2852*) (Holden et al., 2009) encoding putative DGCs. However, the H111 genome does not have this duplication (Carlier et al., 2014), hence the corresponding H111 homolog is termed *bcal2852* here.

Besides their c-di-GMP-metabolizing domains, twenty proteins have additional sensor domains, such as phospho-receiver domains (REC) and domains associated with nucleotide (GAF) or ligand binding (PAS, CBS) (Ponting and Aravind, 1997; Ho et al., 2000; Hoch, 2000; Ereno-Orbea et al., 2013). Eleven proteins are predicted to be integrated into the cytoplasmic membrane and possess membrane-associated sensor domains (MASE) formed by multiple transmembrane helices, periplasmic ligand-binding domains (CACHE, CHASE), or domains necessary for formation of disulfide bonds (CSS) (Anantharaman and Aravind, 2000; Galperin et al., 2001; Nikolskaya et al., 2003; Hengge et al., 2015), whereas intramolecular signal transduction from, e.g., periplasmic sensor domains to cytoplasmic output domains can be facilitated through HAMP domains (Aravind and Ponting, 1999). Although the Bcal0652, Bcal1100, and Bcal2749 protein sequences are longer than solitary EAL domains, we failed to identify additional domains. However, sensor domains are most likely present here, too, and might be identified with future domain database versions of Pfam or CDD. In contrast to *E. coli* (e.g., PdeH), *Salmonella enterica* (e.g., YdiV and YhjH), and *Klebsiella pneumoniae* (e.g., KPN_03274), the *B. cenocepacia* genome does not code for a protein consisting of a solitary, conserved ∼250 AAs long EAL domain only, suggesting that none of the c-di-GMP metabolizing proteins underlies transcriptional and translational regulation only. Besides the composite proteins Bcal0621 and CdpA, which have in addition to their degenerated EAL- or GGDEF- domains, respectively, a second conserved c-di-GMP associated domain and are therefore most likely enzymatically active, the solitary proteins Bcal2749 and Bcal3188 harbor strongly degenerated EAL domains (Figure 1). However, a regulatory function as it has been described for BluF and CsrD in *E. coli* or LapD in *Pseudomonas fluorescens* and *P. putida* (Gjermansen et al., 2005, 2010; Suzuki et al., 2006; Newell et al., 2009; Tschowri et al., 2009) seems likely for Bcal2749 and Bcal3188, particularly as both are conserved among different *Burkholderia* species (Plumley et al., 2017).

Using a recently published method developed by our group (Fazli et al., 2015), we constructed a mutant library with individual gene deletions of the GGDEF/EAL/HD-GYP domain encoding genes in *B. cenocepacia* H111, and investigated the resulting strains for c-di-GMP dependent phenotypes. Several studies have suggested that the lack of a single GGDEF/EAL/HD-GYP domain protein does not necessarily change global intracellular levels of c-di-GMP, indicating that most enzymes interact with specific effectors and affect distinct c-di-GMP-regulated phenotypes without affecting the total cellular c-di-GMP pool (Merritt et al., 2010; Massie et al., 2012; Sarenko et al., 2017). Recent studies have suggested that molecular regulation takes place at a local level *via* direct protein-protein interactions or gradients and closed pools providing locally defined c-di-GMP concentrations (Abel et al., 2011; Lindenberg et al., 2013; Dahlstrom et al., 2015). In addition, these studies have provided evidence that c-di-GMP regulated phenotypes, such as colony morphology, congo red binding and motility, are not necessarily correlated with changes in global cellular c-di-GMP levels (Dahlstrom et al., 2015; Sarenko et al., 2017). Therefore, in the present study we focused on assessing the phenotypic changes of mutants defective of putative c-di-GMP metabolizing proteins in *B. cenocepacia*, rather than determining c-di-GMP concentrations of whole-cell extracts of our mutants.

### The Swimming Phenotype of Distinct DGC and PDE Mutants Suggests That c-di-GMP Is a Negative Regulator of Motility in *B. cenocepacia*

In many bacteria, high intracellular levels of c-di-GMP has been shown to inhibit motility, by either negatively regulating flagellar gene expression or binding to the molecular break, YcgR, hence interfering with flagellar rotation (Ryjenkov et al., 2006; Boehm et al., 2010; Römling et al., 2013). Recent findings in *B. pseudomallei* (Plumley et al., 2017), *B. lata* (Jung et al., 2017), *B. cepacia* (Ferreira et al., 2013), and *B. cenocepacia* (Deng et al., 2012; Kumar et al., 2018) suggest that the modulation of c-di-GMP levels in *Burkholderia* sp. affects swimming motility in semi-solid agar.

**FIGURE 2:**
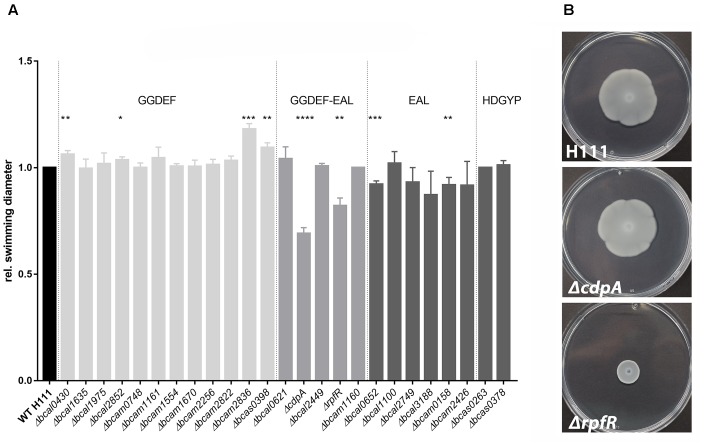
Motility of *B. cenocepacia* H111 and PDE/DGC mutants in semi-solid medium. **(A)** Swimming motility. Overnight cultures of *B. cenocepacia* H111 and mutant derivatives were adjusted to OD_600_ 4 and inoculated into swim plates. Plates were incubated at 37°C and the relative swimming diameter was measured after 10 h. Strains with disruption of genes encoding GGDEF-only proteins are highlighted in light gray, composite GGDEF-EAL protein mutants in gray, EAL-only and HD-GYP-domain protein mutants in dark gray, respectively. Data was normalized to wild type results and shows mean swimming diameter and standard derivations of three individual experiments. Asterisks indicate *P* < 0.05 with unpaired *t*-test. **(B)** Swarming motility. Overnight cultures of *B. cenocepacia* H111 and mutant derivatives were adjusted to OD_600_ 4.0 and inoculated on top of a swarm plate. Plates were incubated at 37°C and pictures were captured after 24 h. Data shown here are representatives of three independent experiments.

In the present study, we provide evidence for a negative effect of elevated c-di-GMP levels on motility in *B. cenocepacia* H111. While the deletion of the GGDEF domain encoding genes resulted in increased swimming motility in semi-solid medium up to 18% (*Δbcam2836*), the deletion of several EAL domain genes (e.g., *Δbcal0652* and *Δbcam0158*) caused a slightly impaired motility phenotype (Figure 2A). However, the deletion of *rpfR* (*bcam0580*) or *cdpA (bcal1069)* resulted in the strongest reduction in swimming diameter (18 and 31% compared to the wild type level) (Figure 2A). Similar results were reported for a *bcal1069* mutant in *B. cenocepacia* K56-2 in synthetic CF sputum medium and homolog gene mutations in *B. pseudomallei*, leading to renaming of *bcal1069* to *cdpA* (Lee et al., 2010; Kumar et al., 2018). In contrast to swimming motility in semi-solid medium, swarming motility can be observed in medium with higher viscosity and requires hyper-flagellation as well as production of extracellular products, such as surfactants, proteins, or polysaccharides (Harshey, 1994). However, CdpA′s stimulating effect on motility seems to be restricted to swimming motility, as swarming motility was only affected in the *rpfR* mutant under the conditions tested (Figure 2B). The DGC/PDE mutants showed wild-type levels of pilus-dependent twitching motility under the conditions tested in the present study (data not shown).

Taken together, our data showed that the deletion of specific genes encoding GGDEF- or EAL-domain proteins resulted in altered swimming motility of *B. cenocepacia* H111. Increased swimming motility of the *bcam2836* mutant, which is deleted for a gene that has only a GGDEF domain, and reduced swimming motility of the *cdpA* and *rpfR* mutants, for which increased c-di-GMP levels have been reported in *B. cenocepacia* and *B. pseudomallei* (Lee et al., 2010; Deng et al., 2012; Plumley et al., 2017; Kumar et al., 2018), suggest that swimming motility is negatively affected by c-di-GMP in *B. cenocepacia*. However, in contrast to what has been shown for *B. pseudomallei*, where swimming motility was completely abolished by the *cdpA* mutation (Lee et al., 2010), none of our *B. cenocepacia* DGC/PDE mutants displayed a total loss of swimming motility.

### *rpfR* Deletion Stimulates Pellicle Formation by *B. cenocepacia* Under Multiple Conditions

While bacterial motility is negatively regulated by high intracellular levels of c-di-GMP, the synthesis of extracellular matrix substances resulting in biofilm formation is usually stimulated by the second messenger. In *B. cenocepacia*, the Bep polysaccharide, synthesized by a multi-protein complex encoded by the *bepA-L* locus, is positively regulated by c-di-GMP and functions as an extracellular matrix component in flow-cell biofilms, pellicles, and macrocolonies, especially in cells with artificially increased c-di-GMP levels (Fazli et al., 2011, 2012, 2017). While the appearance of flow-cell biofilms and macrocolonies depends also on additional matrix components (Huber et al., 2002; Conway et al., 2004; Yakandawala et al., 2011; Inhulsen et al., 2012), the formation of pellicles at the air-liquid interface of liquid cultures was shown to be a Bep-specific phenotype (Fazli et al., 2011, 2012).

**FIGURE 3:**
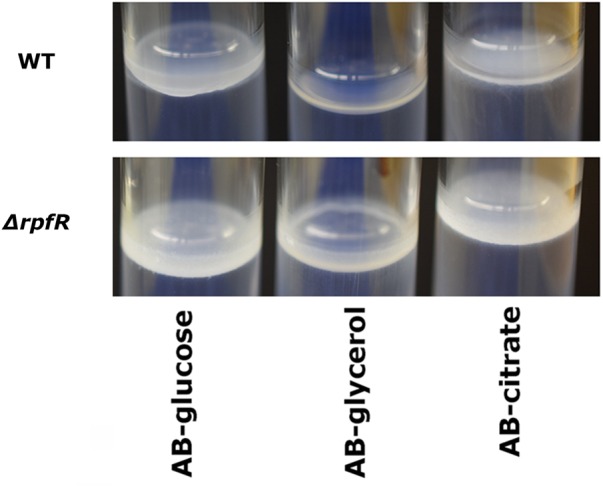
Pellicle formation of *B. cenocepacia* H111 and the *rpfR* mutant in static batch cultures. Wild type and mutants were grown at 30°C in ABnoNaCl supplemented with 10 mM glucose, 10 mM glycerol or 10 mM citrate, respectively, in glass tubes and sealed with parafilm to diminish evaporation but allow air exchange. Pictures of pellicles were captured after 72 h.

As Bep expression is stimulated by high levels of c-di-GMP, the question arose which DGC stimulates its synthesis through c-di-GMP production and which PDE represses its synthesis through degradation of the signal molecule. Therefore, we performed pellicle biofilm formation assays with all 25 DGC/PDE mutants and the wild type strain under different medium and temperature conditions. None of the twelve GGDEF gene deletion mutants were impaired in pellicle formation (data not shown), excluding the existence of a Bep-specific DGC. Furthermore, the EAL and HD-GYP gene deletion mutants and four out of five GGDEF-EAL mutants did not show altered pellicle formation. However, the *rpfR* mutant developed a pellicle already after 48 h of incubation at 30°C in minimal medium, which is significantly faster than the wild type. Prolonged incubation led to pellicle formation by the wild type, too, but the pellicle formed by the *rpfR* mutant was significantly thicker than the wild type pellicle after 72 h incubation (Figure 3). When incubated at room temperature in LBnoNaCl, pellicle formation after 120 h was increased in the *rpfR* mutant, too (data not shown). Therefore, the composite protein RpfR appears to be a negative regulator of the formation of pellicle biofilms. However, we did not identify a single DGC as responsible for delivering c-di-GMP to this process.

### RpfR and Bcal2449 Modulate the Morphology of *B. cenocepacia* Macrocolonies

**FIGURE 4:**
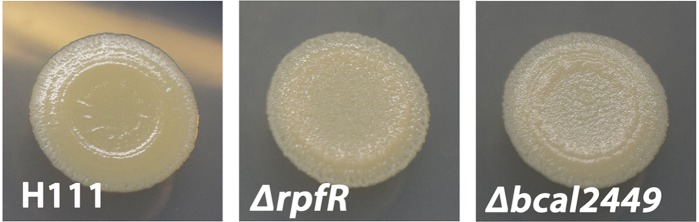
Macrocolony formation of the *B. cenocepacia* wild type, and *rpfR* and bcal2449 mutants on solid agar. *B. cenocepacia* H111 and mutant derivatives were grown on ABnoNaCl agar supplemented with 1% glucose and incubated 5 days, after which images were acquired.

*Burkholderia* spp. in biofilms were shown to produce multiple extracellular polysaccharides (e.g., Bep, cellulose and cepacian) and protein-based matrix components (e.g., BapA, fimbria, lectins) (Ferreira et al., 2011; Yakandawala et al., 2011; Fazli et al., 2012; Inhulsen et al., 2012; Cescutti et al., 2013). A correlation between colony morphology and the production of extracellular matrix components has been reported for several bacterial species, including *B. cenocepacia* (e.g., Fazli et al., 2012). To investigate whether some of our DGC/PDE mutants displayed changed matrix production, we used solid agar plates spot inoculated with cell culture and allowed them to develop into macrocolonies for 5 days at 37°C. In contrast to the wild type and the majority of the GGDEF/EAL/HD-GYP mutants, the *ΔrpfR* and *Δbcal2449* mutants developed dry and structured colonies, suggesting that these mutants secrete increased amounts of extracellular matrix material or that it has an altered composition (Figure 4 and Supplementary Figure S1). We note that unlike the *ΔrpfR* mutant the *Δbcal2449* mutant did not display increased pellicle formation, indicating that the matrix produced by the *Δbcal2449* mutant differ from that produced by the *ΔrpfR* mutant. The RpfR and Bcal2449 proteins have conserved GGDEF and EAL domains and therefore can potentially act as DGC or PDE, depending on intra-molecular regulation or interaction with protein partners. Our data suggest that under the conditions tested RpfR and Bcal2449 both have a negative effect on the production of one or more matrix components.

### RpfR Regulates *B. cenocepacia* Biofilm Formation in Microtiter Plates

Growth of bacteria in the wells of microtiter plates followed by staining of attached cells with CV serves as an established method for quantification of biofilm formation. BIOLOG phenotypic microarray plates (BIOLOG, Inc.) allow growth of bacteria under 95 different medium conditions in parallel, and was used recently to elucidate the effect of environmental conditions on the regulation of c-di-GMP dependent biofilm formation in *P. fluorescens* (Dahlstrom et al., 2018). In addition, recent publications have shown a medium and temperature dependent motility and biofilm phenotype for c-di-GMP mutants in *B. cenocepacia*, *B. pseudomallei*, and *P. fluorescens* (Plumley et al., 2017; Dahlstrom et al., 2018; Kumar et al., 2018). Besides transcriptional and translational regulation, DGCs and PDEs can be regulated at the post-translational level, and environmental stimuli may be perceived by sensor domains. Therefore, individual DGCs and PDEs might respond individually to different environmental signals, such as carbon sources.

Using BIOLOG PM-1 phenotype microarray plates, we assessed the effect of 95 different carbon sources on the biofilm formation properties of our GGDEF/EAL/HD-GYP mutants. After 24 h, detectable cell growth (OD_600_ > 0.2) for the *B. cenocepacia* H111 wild type and mutants was observed for 65 carbon sources, while CV binding was measurable for the wild type and mutants for 90 carbon sources (OD_590_ > 0.25). In total, 52 microtiter wells showed both bacterial growth and visible CV binding after medium and planktonic cells had been discarded. Among these, we focused on the carbon sources which altered CV values by at least 20 percent when compared to the wild type in at least one mutant and, in addition, included the medium used for macrocolony and pellicle experiments, resulting in 22 different media. We performed standard biofilm assays in microtiter plates and quantified cell attachment on microtiter wells as well as pegs dipped into the wells.

**FIGURE 5:**
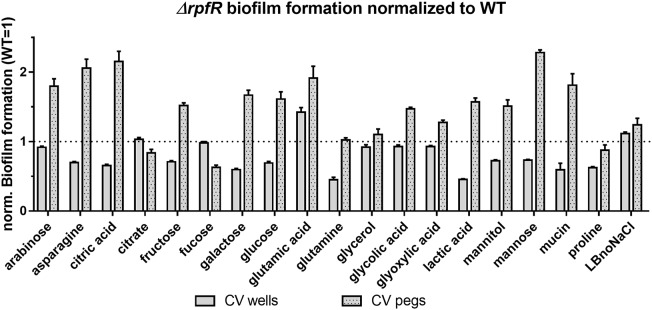
Biofilm formation in microtiter plates by the *B. cenocepacia rpfR* mutant under various medium conditions. Cells were grown in ABnoNaCl supplemented with carbon sources as indicated (see material and methods for concentrations) under static conditions at 37°C for 24 h. Subsequently, the amount of biofilm on the wells and on the pegs, respectively, was determined via a CV staining assay. Graphs show OD_590nm_ values of CV bound by the *ΔrpfR* mutant normalized to values obtained from the wild type. Error bars indicate standard deviations of 4 replicates per strain, and data shown here are representatives of three independent experiments.

Compared to the wild type, the *ΔrpfR* mutant showed altered biofilm formation under most culture conditions (Figure 5 and Supplementary Figure S8). However, while *ΔrpfR* biofilm formation on the pegs was increased, attachment to the microtiter wells was decreased when grown with asparagine, citric acid, fructose, galactose, glucose, lactic acid, mannitol, mannose, and mucin as the carbon source. In contrast, growth with fucose, glutamine or proline altered only one form of biofilm formation, and growth on glutamic acid enhanced attachment to both the pegs and the wells. Among the remaining 24 strains tested, the GGDEF mutants *Δbcam2836* and *Δbcas0398* showed slightly enhanced attachment under several conditions to both the pegs and the wells (e.g., *Δbcas0398* in fucose media), and for the GGDEF-EAL mutant *Δbcam1160* reduced attachment to the wells was observed in medium supplemented with glycolic acid and glyoxylic acid (Supplementary Figures S2–S7). These two compounds did not change attachment patterns of the *rfpR* mutant. In contrast to our initial results from the BIOLOG plates, cell growth and detectable amounts of CV binding could not be observed in medium supplemented with lactose, serine and tyramine, and the results with these media are therefore not shown.

Taken together, our results suggest that RpfR is the master regulator of biofilm formation in microtiter plates under most conditions, although Bcam2836, Bcas0398, and Bcam1160 seem to affect biofilm formation under some conditions. Our results suggest that *B. cenocepacia* biofilm formation on pegs is positively regulated by c-di-GMP, whereas biofilm formation on wells (the “standard” microtiter plate assay) is negatively regulated by c-di-GMP, suggesting that these two forms of biofilm formation differ, and are under different regulatory mechanisms in *B. cenocepacia*.

### RpfR Regulates *B. cenocepacia* Biofilm Formation in Flow-Cells

Similar to microtiter plates, flow-cells provide an abiotic surface for cell attachment and formation of biofilms. However, in contrast to microtiter plates, flow-cell incubation guarantees constant nutrient supply due to a continuous flow of fresh medium as well as removal of metabolic waste products, used medium and cell debris. In addition, the maturing biofilm is exposed to mild shear forces, without the risk of desiccation due to medium evaporation. Prior work in our group has demonstrated that *B. cenocepacia* biofilm formation in flow-cells is positively regulated by c-di-GMP (Fazli et al., 2011, 2012).

**FIGURE 6:**
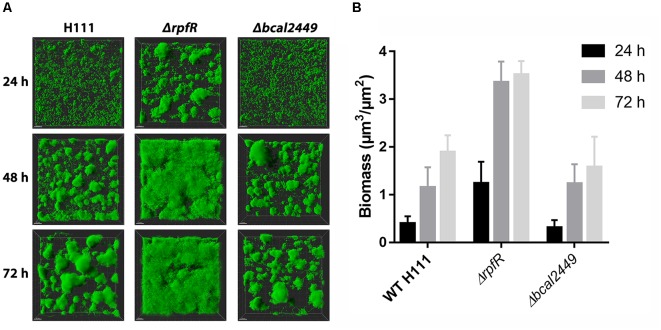
Biofilm formation in flow-cells by *B. cenocepacia* H111 and the *rpfR* and *bcal2449* mutants. **(A)** Confocal laser scanning micrographs of 3-day-old biofilms formed by *gfp*-expressing *B. cenocepacia* strains. The images show top-down easy3D views **(B)** Comstat analysis of biomass for 1, 2 and 3 day old biofilms of wild type and mutants. Figure shows mean and standard derivations of six CLSM images per strain and time point.

To assess the effect of individual GGDEF, EAL, or HD-GYP deletions, we tested biofilm formation phenotypes of our 25 GGDEF/EAL/HD-GYP mutants and the corresponding wild type in flow-cells over a time frame of 3 days. Most of the mutants did not display altered biofilm formation in the flow-cells compared to the wild type (Supplementary Figure S9). However, similar to the results obtained from biofilm assays in microtiter plates, the *rpfR* mutant displayed an altered biofilm formation phenotype (Figure 6 and Supplementary Figure S9). The *rpfR* mutant formed compact, spherical microcolonies after 24 h, which were absent in the biofilms formed by the wild type and the other DGC/PDE mutants. After 48 h the *rpfR* mutant cells had colonized the entire substratum with a thick matt-like biofilm, which appeared smooth at the surface, but showed denser structures in underlying layers. Notably, the biomass of the *ΔrpfR* biofilms was increased in comparison to the biomass of the wild type biofilms at all time points (Figure 6B). In contrast to the case of macrocolony formation (Figure 4), the *bcal2449* mutant did not display altered biofilm formation in flow-cells (Supplementary Figure S9).

Taken together, our results suggest that RpfR negatively regulates the formation of microcolonies in young biofilms and development into mature biofilms during prolonged incubation in flow-cells. We did not identify a single DGC as responsible for delivering c-di-GMP to stimulate biofilm formation in flow-cells.

### Bcal2449 Is a Major Regulator of *B. cenocepacia* Virulence

**FIGURE 7:**
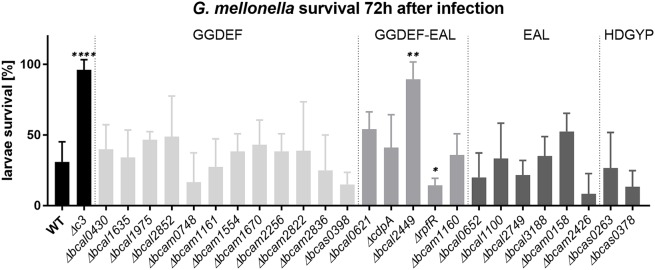
Virulence of *B. cenocepacia* H111 and PDE/DGC mutants in *G. mellonella*. Ten larvae were infected with 10.000–20.000 *B. cenocepacia* wild type or mutants, and killing was observed over 3 days. Data presented here show mean and standard deviations of larvae survival of at least 3 independent experiments per strain 72 h after infection. Knockout mutants of genes coding for GGDEF-only proteins are highlighted in light gray, those encoding composite GGDEF-EAL proteins in gray, EAL-only proteins and HD-GYP-domain proteins in dark gray, wild type and *Δc3* control in black, respectively. Asterisks indicate *P* < 0.05 with unpaired *t*-test with wild type control.

**FIGURE 8:**
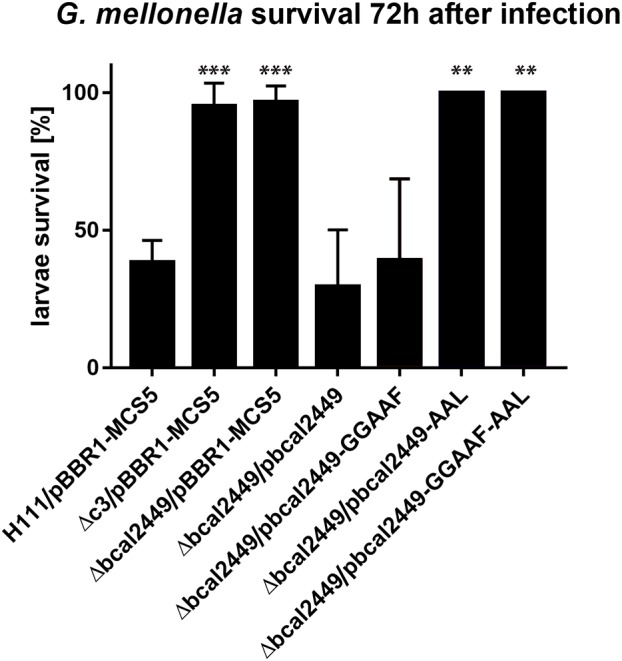
Effect of mutations in the EAL and GGDEF domain of Bcal2449 on virulence of *B. cenocepacia* in *G. mellonella*. Ten larvae were infected with 10.000–20.000 *B. cenocepacia* wild type or mutants harboring pBBR1 plasmid derivatives as indicated, and larvae killing was observed over 3 days. Data presented here show mean and standard deviations of larvae survival of at least 3 independent experiments per strain and the corresponding wild type control 72 h after infection. Asterisks indicate *P* < 0.05 with unpaired *t*-test with wild type control.

Besides motility and biofilm formation, c-di-GMP is known to regulate virulence in a number of bacteria (Römling et al., 2013; Jenal et al., 2017; Hall and Lee, 2018), including *B. cenocepacia*, where high c-di-GMP levels appear to be associated with impaired virulence (Schmid et al., 2017). We determined the virulence phenotype of each individual GGDEF, EAL, or HD-GYP mutant in larvae of the Greater Wax Moth *G. mellonella* (Figure 7). Interestingly, we found that the deletion of the GGDEF-EALxR-containing Bcal2449 protein almost abolished *B. cenocepacia* virulence (Figure 7). The virulence was restored when *bcal2449* was expressed *in trans* from the plasmid pBBR1-*bcal2449* (Figure 8). Larvae killing was strictly dependent on a conserved EAL-motif, as the complementation of the *bcal2449* mutant strain with a mutated EAL domain (*Δbcal2449*/pBBR1-bcal2449-AAL and *Δbcal2449*/pBBR1-bcal2449-GGAAF-AAL) failed to restore the virulence (Figure 8), suggesting that a decreased c-di-GMP level mediated by the Bcal2449 protein is required for virulence. In contrast, bacteria expressing a *bcal2449*-variant with a mutated GGDEF domain (*Δbcal2449*/pBBR1-*bcal2449*-GGAAF) were as virulent as the complemented mutant (Figure 8). Besides, our screen suggested that infection with the *rpfR* or *bcam2426* mutants resulted in reduced larval survival in comparison to the wild type (Figure 7), although the results for the *rpfR* and *bcam2426* mutants are not as pronounced as the result obtained for the *bcal2449* mutant. An attenuated virulence phenotype of *ΔcdpA* was not observed in the present study. Therefore, we suggest that CdpA only acts on motility in *B. cenocepacia* and does not play a major role in other c-di-GMP regulated processes, in contrast to its homolog in *B. pseudomallei* (Lee et al., 2010).

Taken together, our results suggest a major role for the composite protein Bcal2449 in *B. cenocepacia* virulence, which is dependent on a functional EAL domain, suggesting that a local decrease in c-di-GMP mediated by the Bcal2449 protein is required for virulence in the *G. mellonella* larvae model.

## Discussion

The intracellular signaling molecule c-di-GMP is synthesized and degraded by GGDEF- and EAL-/HD-GYP-domain proteins, respectively, and regulates a variety of cellular processes in a wide range of bacteria, including *B. cenocepacia*. This study represents the first detailed analysis of the function of all 25 GGDEF-/ EAL- and HD-GYP-domain proteins encoded by the *B. cenocepacia* H111 genome, focusing on their individual effects on motility, biofilm formation and virulence.

RpfR, a multi-domain protein consisting of a BDSF-binding PAS-, a GGDEF- and an EAL-domain, was previously shown to regulate motility, protease activity, biofilm formation and virulence of *B. cenocepacia* (Deng et al., 2012; Jung et al., 2017; Schmid et al., 2017). Our data presented in this report suggest that RpfR is a key regulator of c-di-GMP signaling in *B. cenocepacia*. Previous studies revealed a reduced ability of biofilm formation in the wells of microtiter plates for *rpfR* mutants (Deng et al., 2012; Suppiger et al., 2016; Yang et al., 2017). Striking results, considering that deletion of *rpfR* also resulted in reduced motility and increased intracellular c-di-GMP levels. Our analysis verified decreased biofilm formation of the *rpfR* mutant in the wells of microtiter plates, however, we found that biofilm formation on polystyrene pegs, in flow-cells and in pellicles was increased for the *rpfR* mutant, suggesting that high levels of c-di-GMP, due to a lack of this key-PDE, positively regulate biofilm formation by *B. cenocepacia* under various conditions.

The CepIR quorum sensing system appears to control biosurfactant production in *B. cenocepacia* (Huber et al., 2001) and is, in turn, modulated by the RpfRF system (Schmid et al., 2012). Hence, RpfR affects both flagellar gene expression (BDSF regulated) and indirectly biosurfactant production (AHL regulated) and therefore swimming and swarming motility (Lee et al., 2010). Accordingly, we observed reduced swimming motility for the *rpfR* mutant and, in addition, also for the *cdpA* mutant. In *B. pseudomallei* swimming motility is completely abolished in a *cdpA* mutant, and the cells are aflagellate and do not express *fliC* (Lee et al., 2010). In agreement with our results, reduced swimming motility for a *B. cenocepacia cpdA* mutant has also been described by Kumar et al. (2018), and this was found not to be the result of altered flagellin expression or flagellation patterns, suggesting that cellular targets of CdpA-dependent regulation differ among these species. While motility in *B. cenocepacia* is regulated by CdpA in response to arginine and glutamate (Kumar et al., 2018), biofilm formation is not affected under the conditions tested. However, we show that attachment to abiotic surfaces in the presence of glutamate is regulated by RpfR, suggesting that one extracellular stimulus can regulate different phenotypic outputs through independent c-di-GMP cascades.

To analyze the effect of environmental conditions on biofilm formation, we performed biofilm assays in media supplemented with different carbon sources, which we chose with a focus on, inter alia, the conditions likely prevailing in the natural habitats of *B. cenocepacia*, such as soil and water, plant rhizosphere and CF sputum, or as a component of *Bcc* extracellular polysaccharides (Mahenthiralingam et al., 2005; Palmer et al., 2007; Cuzzi et al., 2014; Mongkolrob et al., 2015). We found that RpfR regulates biofilm formation under multiple nutrient conditions, many of them associated with components present in sputum (galactose, glutamine, lactic acid, mucin, proline) (Palmer et al., 2007). In contrast to the *rpfR* mutant, the *bcam1160* mutant showed reduced biofilm formation in response to plant-associated components, such as glycolic and glyoxylic acid, emphasizing the importance of adjacent sensor domains for signal specificity of individual DGCs and PDEs.

In this study, we simultaneously quantified biofilm formation on the surface of microtiter plate wells and biofilm formation on pegs dipped into the wells. We found that biofilm formation of the *rpfR* mutant was increased on the pegs but decreased on the surface of the wells under various conditions. We also assessed biofilm formation of the wild-type and mutants on the surface of the wells in microtiter plates without pegs, and found no difference compared to when pegs were present (data not shown). These results argue against the possibility that the *rpfR* mutant attached and grew better on pegs, and therefore fewer cells grew attached to the surface of the microtiter wells, resulting in simply a redistribution of biomass. Moreover, given that the RpfR protein has been reported to act as a PDE under the conditions of the microtiter tray assay (Deng et al., 2012), our data for biofilm formation on the surface of microtiter wells agrees with former studies, where a correlation between high c-di-GMP levels (either through PDE mutation or plasmid-borne DGC expression) and reduced biofilm formation in microtiter plates has been observed (Deng et al., 2012; Plumley et al., 2017; Schmid et al., 2017; Yang et al., 2017).

Our analysis of macrocolony morphology of the *B. cenocepacia* wild type and DGC/PDE mutants showed that the *rpfR* and *bcal2449* mutants formed more structured colonies than the wild type, indicating that the RpfR and Bcal2449 proteins act as PDEs at 37°C. During long-term infection, strains with mutations within the N-terminal PAS domain of RpfR were isolated (Silva et al., 2016). As mutations affected amino acid residues essential for BDSF-binding, the mutated protein-variant is most likely unable to bind BDSF, resulting in a constantly inactive or unregulated PDE, which may lead to increased biofilm formation in the lungs of the patient. RpfR was also identified as a key player in an evolutionary biofilm model that allows long-term selection for adherence to and dispersal from a plastic bead in a test tube, emphasizing the central role of this regulator in biofilm formation (Traverse et al., 2013).

Strikingly, RpfR, while being a key component in the c-di-GMP network of *B. cenocepacia*, is not fully conserved within the genus *Burkholderia*, raising the question about a homolog PDE with a comparable broad regulon in these species. The genes *bcal2449*, *bcal2749* and *bcal3188* are conserved among *B. cenocepacia*, *B. glumae*, *B. mallei*, *B. pseudomallei*, and *B. thailandensis* (Plumley et al., 2017). The genes *bcal2749* and *bcal3188* code for proteins with solitary, degenerated EAL domains, which most likely do not take direct action in c-di-GMP degradation. However, their wide distribution as well as their mutated active site within the EAL domain (HASxR and GYLxx, respectively) within the genus *Burkholderia* suggests a prominent role in cellular regulation. Moreover, the two domains of Bcal2449 (GGDEF and EAL) are highly conserved. Our study indicates a major role for Bcal2449 in the regulation of virulence, which is dependent on its EAL domain and therefore most likely its PDE activity. This is in agreement with prior studies, indicating that virulence of *B. cenocepacia* is stimulated at low c-di-GMP levels obtained by plasmid-borne expression of a PDE (Schmid et al., 2017). As this effect is restricted to Bcal2449 only and was not observed for other PDEs such as RpfR or CdpA, which have been shown to regulate global intracellular c-di-GMP levels (Lee et al., 2010; Deng et al., 2012), we propose a local mode of regulation on virulence factors mediated by Bcal2449. Further studies will identify these Bcal2449-controled virulence factors, and the molecular regulation and environmental signals modulating Bcal2449 activity presumably *via* its periplasmic Cache domain.

Our study identified individual c-di-GMP-metabolizing proteins, which regulate a specific c-di-GMP-dependent phenotype. Our mutant analysis indicated that the putative DGC Bcam2836 and the PDE CdpA have opposite effects on motility, suggesting that locally high c-di-GMP levels inhibit motility of *B. cenocepacia*. Consistent with a recent publication (Plumley et al., 2017), we found that Bcam2836 regulates biofilm formation under some conditions. In addition, the GGDEF-EAL domain proteins Bcam1160 and Bcal2449 regulate only some c-di-GMP-dependent phenotypes (macrocolony and microtiter plate biofilm formation/ macrocolony biofilms and virulence), suggesting a high specificity within the c-di-GMP network. In contrast, the quorum sensing regulator RpfR modulates multiple c-di-GMP phenotypes and therefore represents an important key-regulator of multiple phenotypes in *B. cenocepacia*.

Collectively, our study demonstrates that c-di-GMP inhibits motility and stimulates biofilm formation by *B. cenocepacia* under many conditions similar to what has been found for many other bacterial species. RpfR seems to be a master regulator of biofilm formation under various conditions, whereas a few of the other DGC/PDEs were shown to affect biofilm formation under specific conditions. In addition, we present evidence that Bcal2449 is a regulator of *B. cenocepacia* virulence, which is dependent on its EAL domain, presumably lowering the level of c-di-GMP and thereby stimulating virulence. However, the virulence factors and extracellular matrix components controlled by Bcal2449 remain to be elucidated. The role of the remaining GGDEF-, EAL-, and HD-GYP domain proteins as well as environmental clues regulating them has to be addressed in future studies.

## Author Contributions

AR and TT-N designed the experiments. AR, MF, NS, RS, and AS performed the experiments. AR, TT-N, LE, and MG interpreted the data. AR wrote the manuscript with input from all authors.

## Conflict of Interest Statement

The authors declare that the research was conducted in the absence of any commercial or financial relationships that could be construed as a potential conflict of interest.
