# Supplementary material for: Key Players and Individualists of Cyclic-di-GMP Signaling in Burkholderia cenocepacia
Source: Front Microbiol. 2019 Jan 10;9:3286. doi: 10.3389/fmicb.2018.03286 (PMC6335245; doi:10.3389/fmicb.2018.03286)
Supplement: Supplementary file 1 [file Data_Sheet_1.PDF]

Supplementary Material

Key players and individualists of cyclic-di-GMP signaling in *Burkholderia cenocepacia*

Anja M. Richter, Mustafa Fazli, Nadine Schmid, Rebecca Shilling, Angela Suppiger, Michael Givskov, Leo Eberl and Tim Tolker-Nielsen*

*** Correspondence:** Tim Tolker-Nielsen: ttn@sund.ku.dk

Supplementary Figures

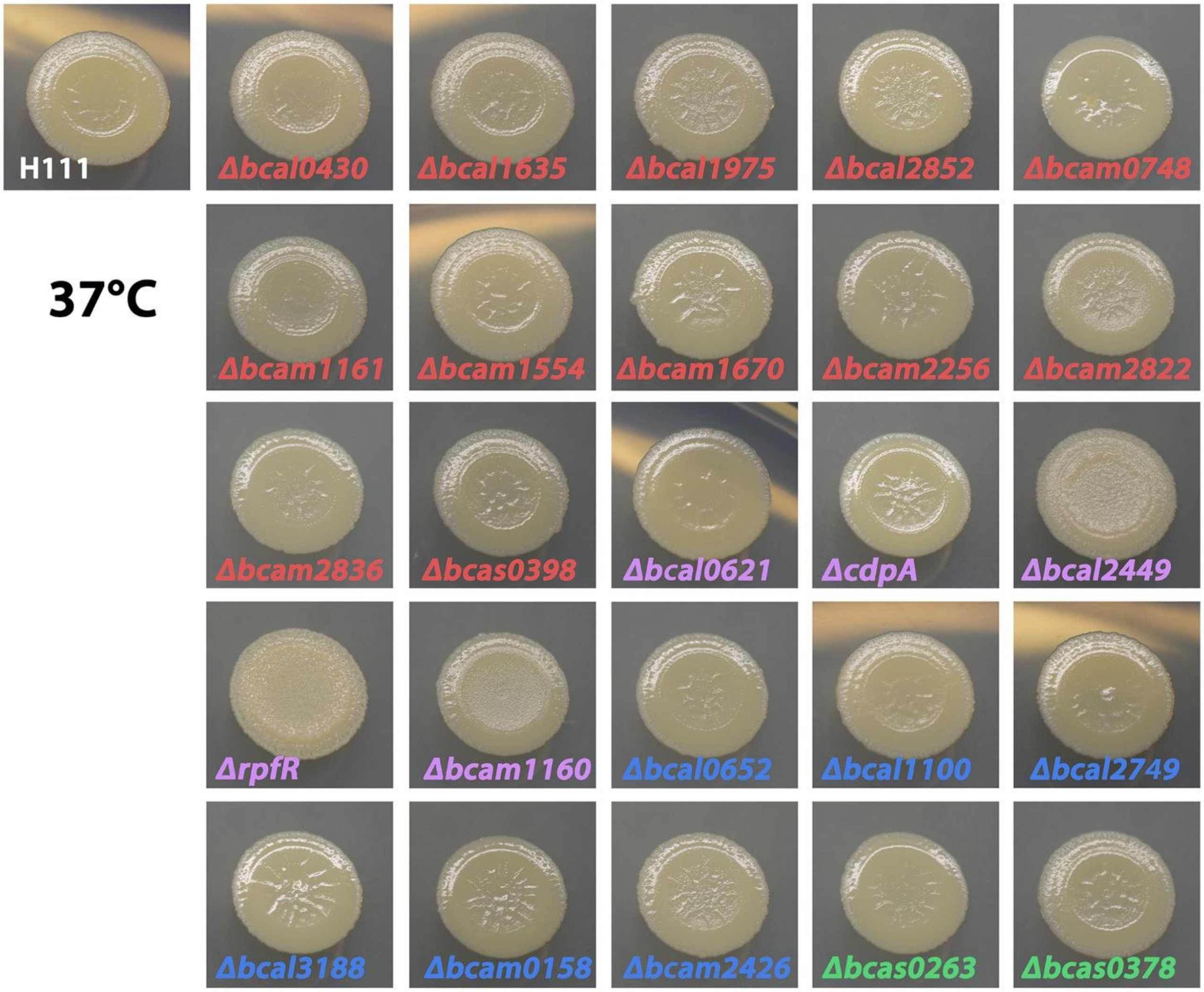

Supplementary Figure 1. Macrocolony formation at 37°C on solid agar. *B. cenocepacia* H111 and mutant derivatives were grown on ABnoNaCl agar supplemented with 1% glucose and incubated at 37°C for 5 days. Knockout mutants of genes coding for GGDEF-only proteins are highlighted in red, those encoding composite GGDEF-EAL proteins in violet, EAL-only proteins in blue and HD-GYP-domain proteins in green, respectively.

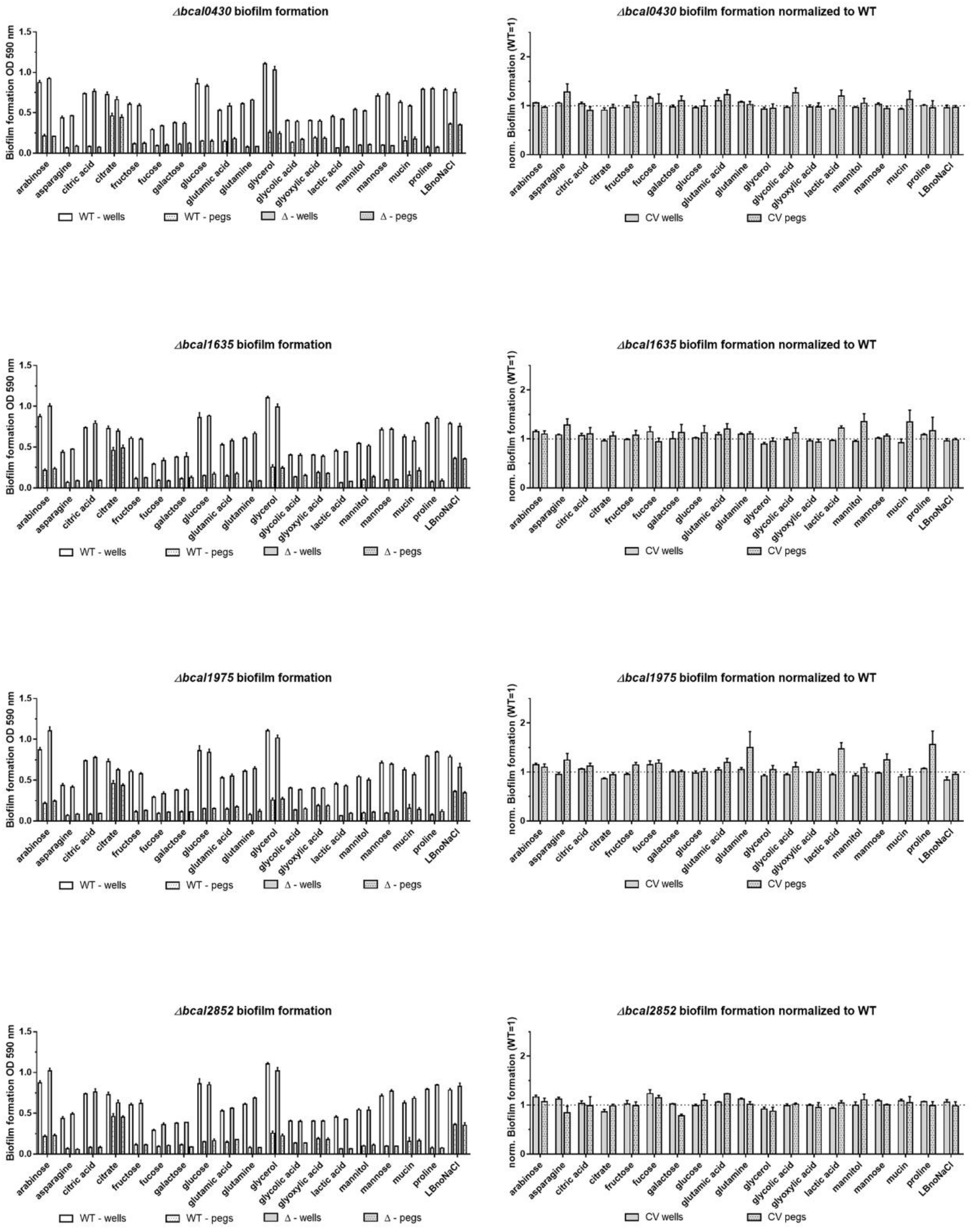

Supplementary Figure 2. Biofilm formation of *bcal0430*, *bcal1635*, *bcal1975* and *bcal2852* mutants in microtiter plates. Cells were grown in ABnoNaCl supplemented with carbon sources as indicated (see material and methods for concentrations) under static conditions at 37°C for 24 h. Subsequently, the amount of biofilm on the wells and on the pegs, respectively, was determined via a CV staining assay. Graphs show OD_{590nm} values of CV bound by wild type and deletion mutants (left panel) and mutant data normalized to values obtained from the wild type (right panel). Error bars indicate standard derivations of 4 replicates per strain, and data shown here are representatives of three independent experiments.

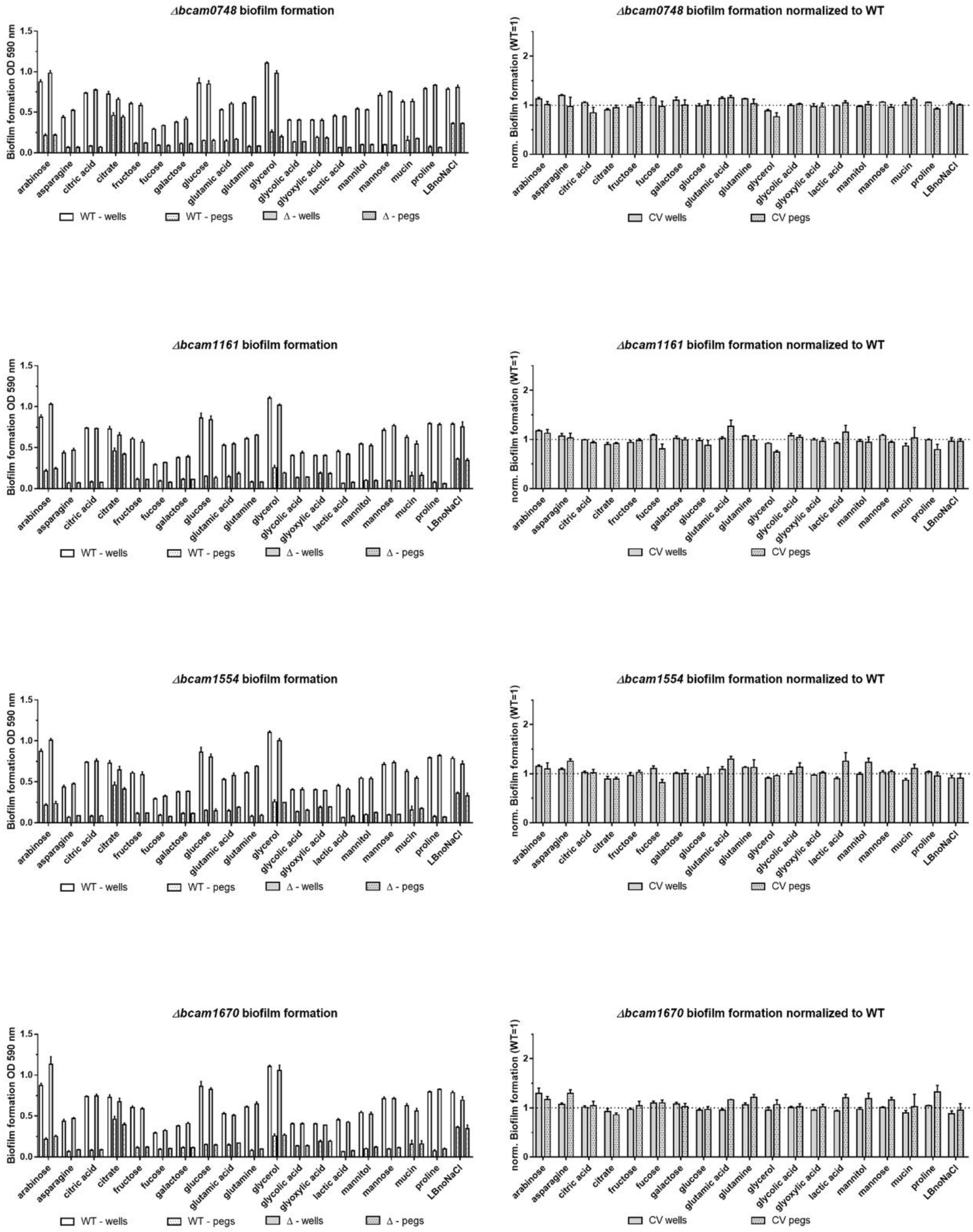

Supplementary Figure 3. Biofilm formation of *bcam0748*, *bcam1161*, *bcam1554* and *bcam1670* mutants in microtiter plates. Cells were grown in ABnoNaCl supplemented with carbon sources as indicated (see material and methods for concentrations) under static conditions at 37°C for 24 h. Subsequently, the amount of biofilm on the wells and on the pegs, respectively, was determined via a CV staining assay. Graphs show OD_{590nm} values of CV bound by wild type and deletion mutants (left panel) and mutant data normalized to values obtained from the wild type (right panel). Error bars indicate standard derivations of 4 replicates per strain, and data shown here are representatives of three independent experiments.

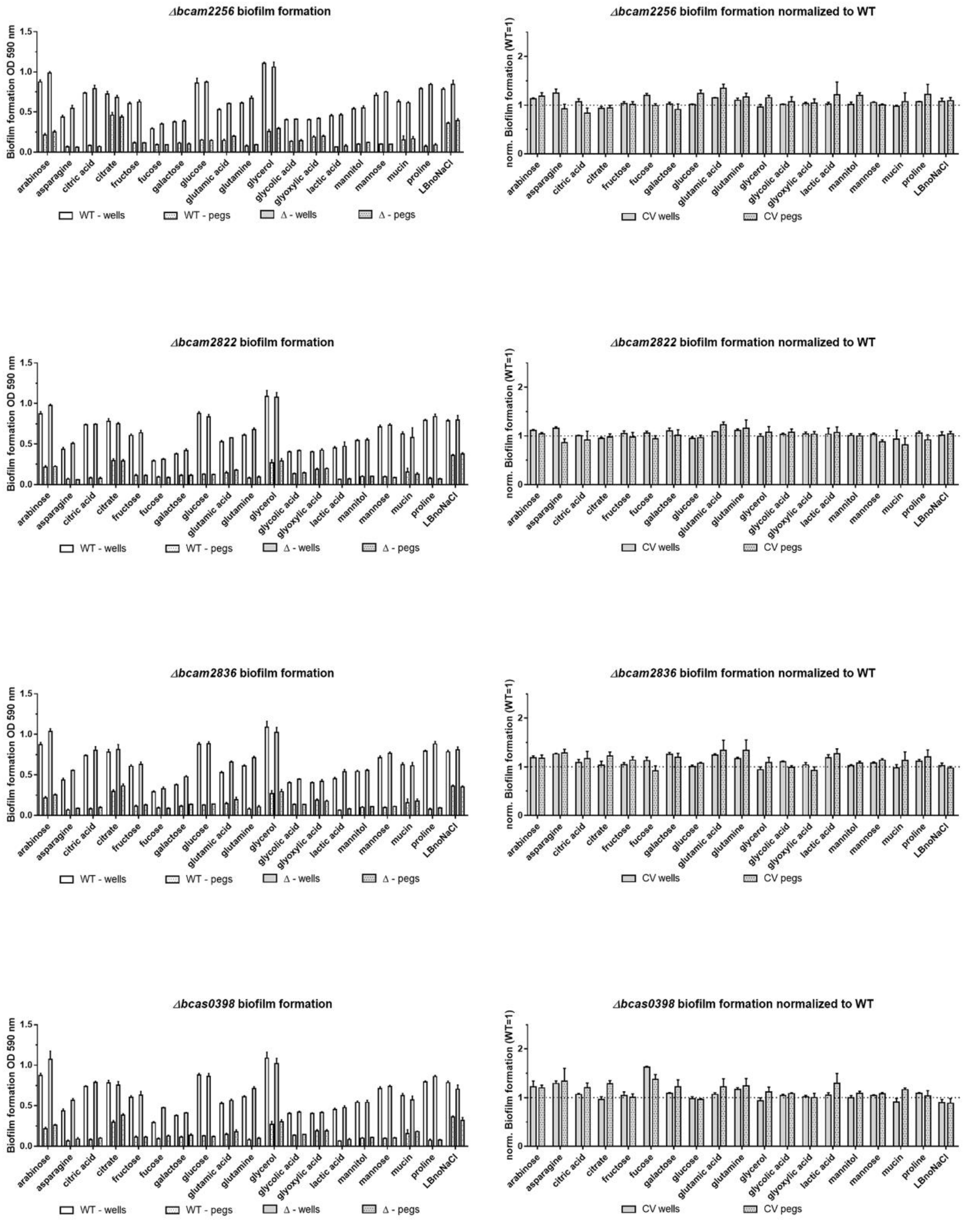

Supplementary Figure 4. Biofilm formation of *bcam2256*, *bcam2822*, *bcam2836* and *bcas0398* mutants in microtiter plates. Cells were grown in ABnoNaCl supplemented with carbon sources as indicated (see material and methods for concentrations) under static conditions at 37°C for 24 h. Subsequently, the amount of biofilm on the wells and on the pegs, respectively, was determined via a CV staining assay. Graphs show OD_{590nm} values of CV bound by wild type and deletion mutants (left panel) and mutant data normalized to values obtained from the wild type (right panel). Error bars indicate standard derivations of 4 replicates per strain, and data shown here are representatives of three independent experiments.

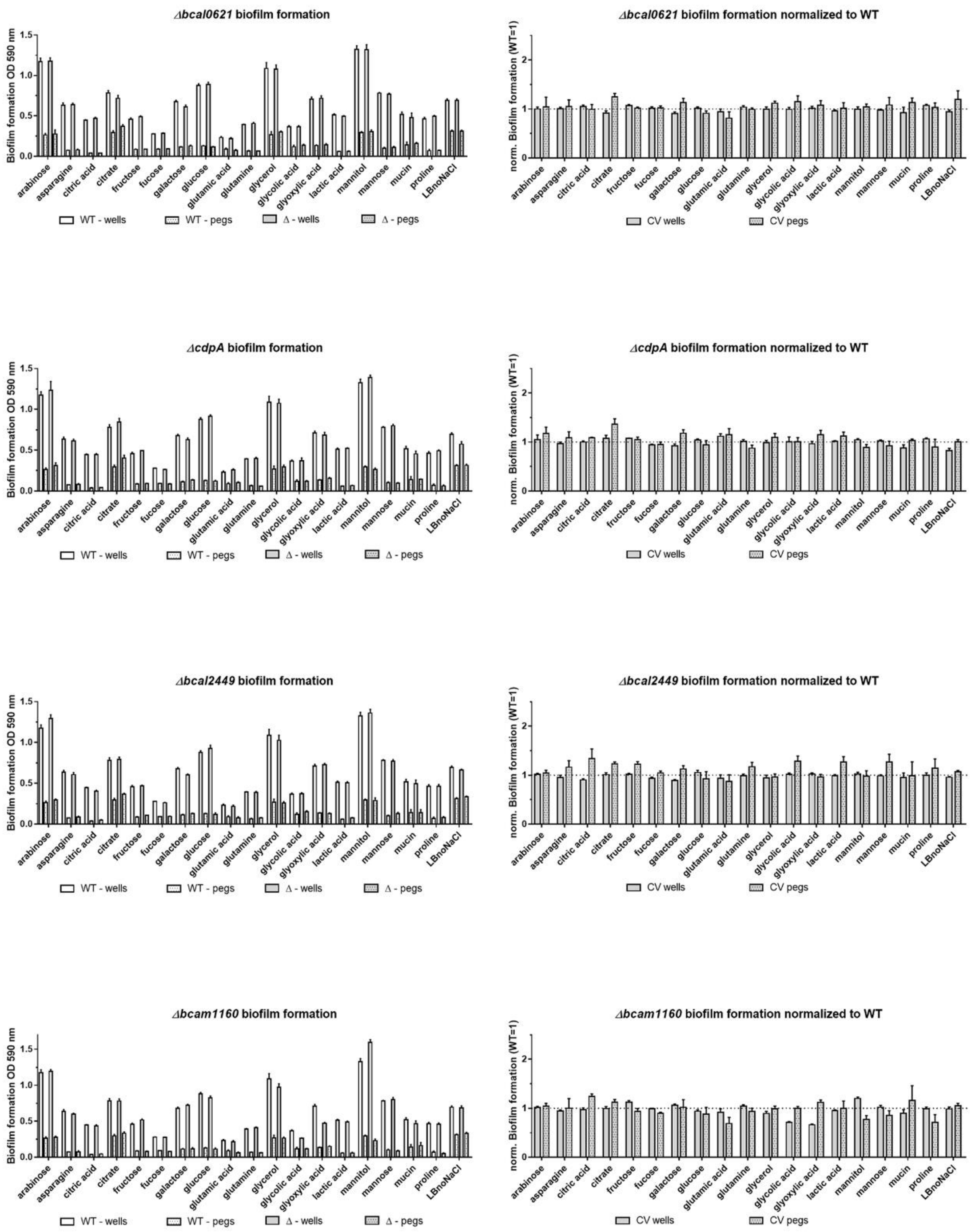

Supplementary Figure 5. Biofilm formation of *bcal0621*, *cdpA*, *bcal2449* and *bcam1160* mutants in microtiter plates. Cells were grown in ABnoNaCl supplemented with carbon sources as indicated (see material and methods for concentrations) under static conditions at 37°C for 24 h. Subsequently, the amount of biofilm on the wells and on the pegs, respectively, was determined via a CV staining assay. Graphs show OD_{590nm} values of CV bound by wild type and deletion mutants (left panel) and mutant data normalized to values obtained from the wild type (right panel). Error bars indicate standard derivations of 4 replicates per strain, and data shown here are representatives of three independent experiments.

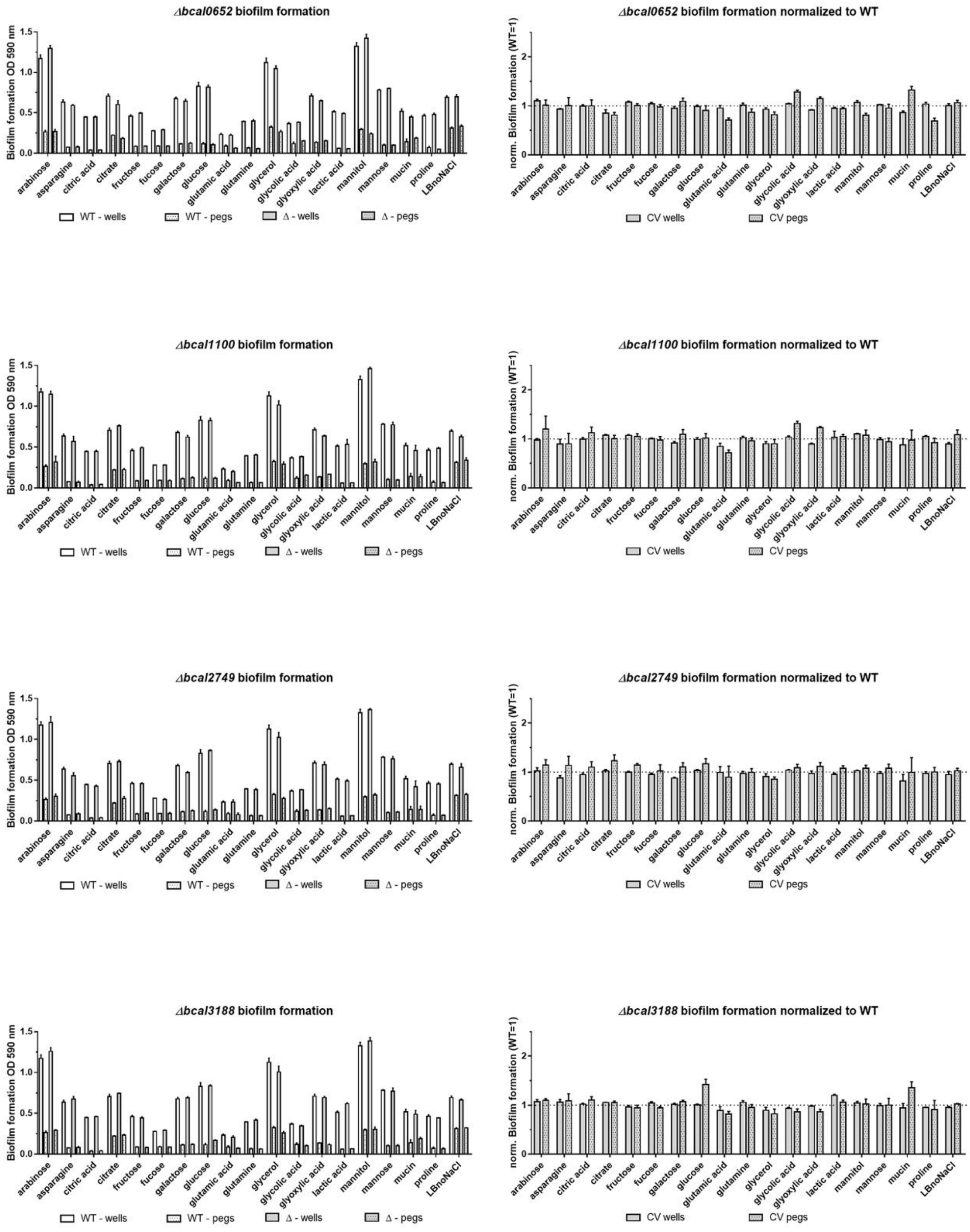

Supplementary Figure 6. Biofilm formation of *bcal0652*, *bcal1100*, *bcal2749* and *bcal3188* mutants in microtiter plates. Cells were grown in ABnoNaCl supplemented with carbon sources as indicated (see material and methods for concentrations) under static conditions at 37°C for 24 h. Subsequently, the amount of biofilm on the wells and on the pegs, respectively, was determined via a CV staining assay. Graphs show OD_{590nm} values of CV bound by wild type and deletion mutants (left panel) and mutant data normalized to values obtained from the wild type (right panel). Error bars indicate standard derivations of 4 replicates per strain, and data shown here are representatives of three independent experiments.

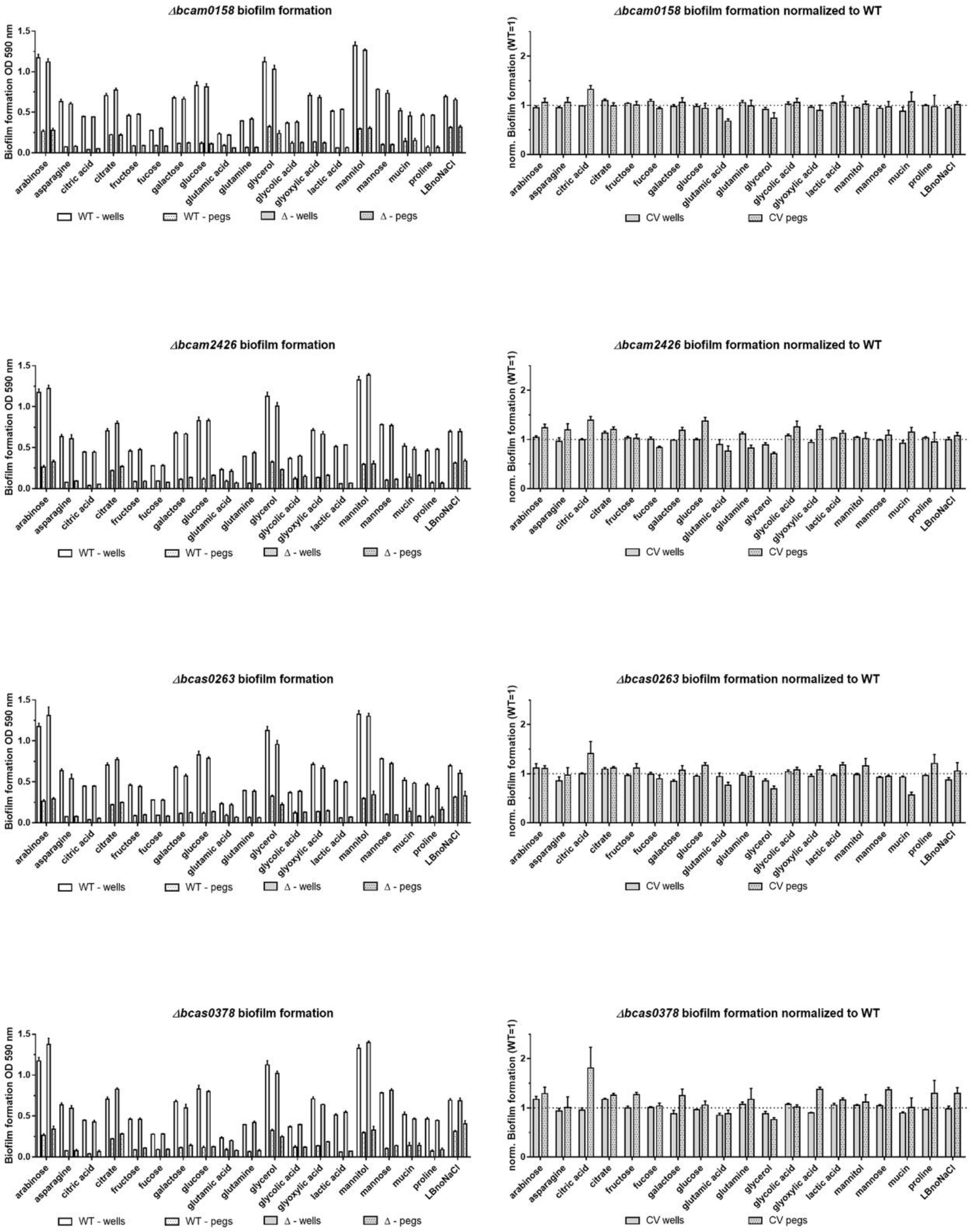

Supplementary Figure 7. Biofilm formation of *bcam0158*, *bcam2426*, *bcas0263* and *bcas0378* mutants in microtiter plates. Cells were grown in ABnoNaCl supplemented with carbon sources as indicated (see material and methods for concentrations) under static conditions at 37°C for 24 h. Subsequently, the amount of biofilm on the wells and on the pegs, respectively, was determined via a CV staining assay. Graphs show OD_{590nm} values of CV bound by wild type and deletion mutants (left panel) and mutant data normalized to values obtained from the wild type (right panel). Error bars indicate standard derivations of 4 replicates per strain, and data shown here are representatives of three independent experiments.

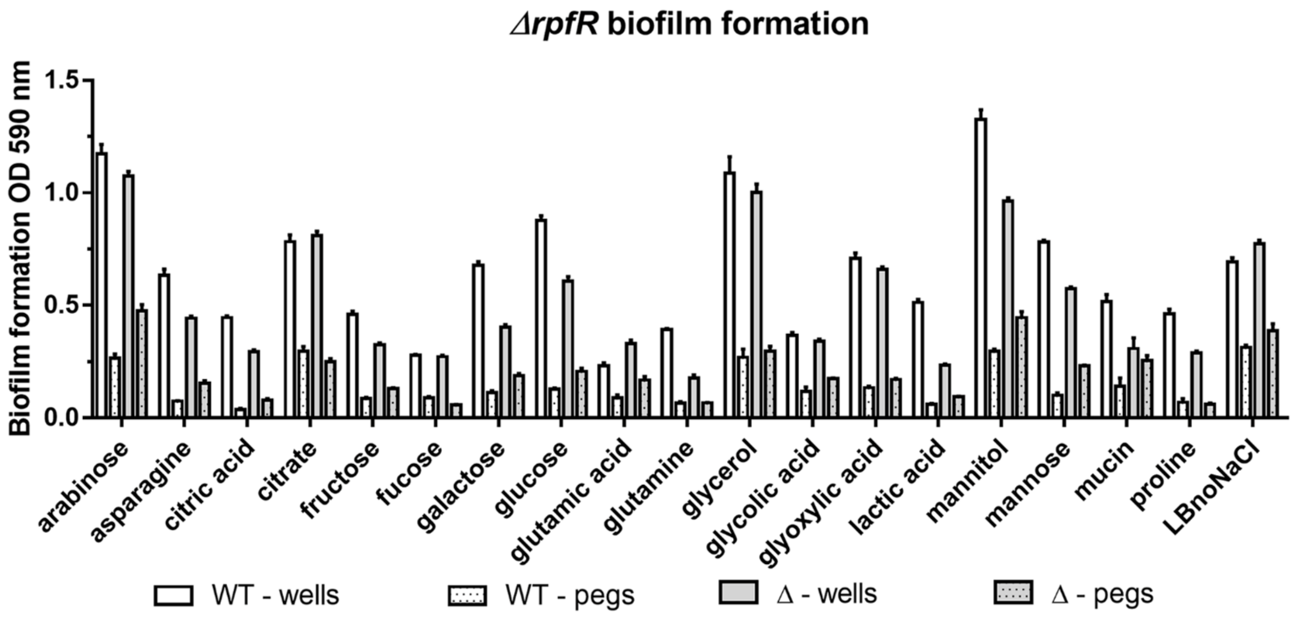

Supplementary Figure 8. Biofilm formation of *rpfR* mutant in microtiter plates. Cells were grown in ABnoNaCl supplemented with carbon sources as indicated (see material and methods for concentrations) under static conditions at 37°C for 24 h. Subsequently, the amount of biofilm on the wells and on the pegs, respectively, was determined via a CV staining assay. Graph shows OD_{590nm} values of CV bound by wild type and deletion mutant (for normalized data see figure 5). Error bars indicate standard derivations of 4 replicates per strain, and data shown here are representatives of three independent experiments.

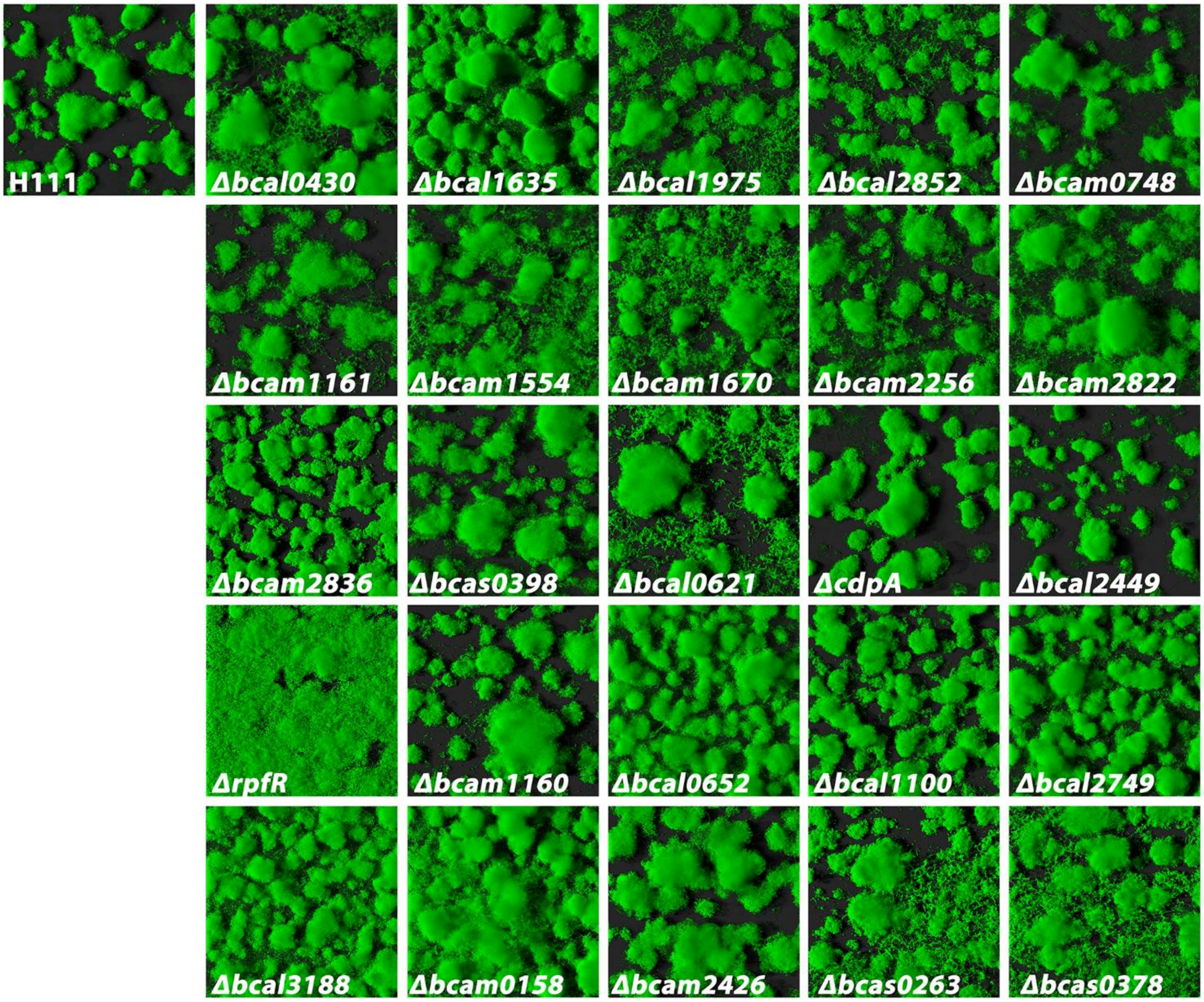

Supplementary Figure 9. Biofilm formation in flow-cells. Confocal laser scanning micrographs of 3-day-old biofilms formed by *gfp*-expressing *B. cenocepacia* strains. Images show top-down easy3D views.
